# Detection of Human Papillomavirus Infection in Cases of Oral Carcinoma Using p16 Immunohistochemistry

**DOI:** 10.7759/cureus.101491

**Published:** 2026-01-13

**Authors:** Thangjam Ruchika Devi, Sunita Y Patil, Kumar Vinchurkar

**Affiliations:** 1 Department of Pathology, Jawaharlal Nehru Medical College, KLE Academy of Higher Education and Research (KAHER), Belagavi, IND; 2 Department of Surgical Oncology, Jawaharlal Nehru Medical College, KLE Academy of Higher Education and Research (KAHER), Belagavi, IND

**Keywords:** human papillomavirus, immunohistochemistry, oral carcinoma, p16, tobacco

## Abstract

Background and aim

Oral cancer is a significant global health concern, with both high prevalence and mortality. In India, oral squamous cell carcinoma (OSCC) represents a large proportion of malignancies. Major risk factors include the use of tobacco and alcohol. High-risk human papillomavirus (HPV) infection has also been recognized as contributing to the development of oral cancers. This study aims to identify HPV infection in oral carcinoma cases using p16 immunohistochemistry (IHC) and to examine its relationship with the histological grading of the tumors.

Materials and methods

A one-year cross-sectional study of N = 50 cases of OSCC was conducted, and histological grading was performed according to the modified Broder’s grading system. All OSCC cases were evaluated for p16 expression. Cases showing p16 positivity and its association with histological grading were analyzed. A p-value of <0.05 was considered statistically significant.

Results

Out of 50 cases, 48% (24/50) were positive for p16, of which 75% (18/24) were male, and 25% (6/24) were female. Among these 24 cases, histological grading showed that 4/24 cases were well differentiated, 19/24 cases were moderately differentiated, and 1/24 case was poorly differentiated. There was no statistically significant association between HPV infection and the histological grading of OSCC using p16 IHC (p-value = 0.304).

Conclusions

The present study revealed p16 positivity in 48% of OSCC cases, suggesting a low prevalence of HPV-infected OSCC in this region. The study also concluded that there is no statistically significant association between p16 expression and the histopathological grading of OSCC. A comparative study of p16 IHC and PCR is recommended for future studies to yield a more accurate and definitive diagnosis.

## Introduction

Oral cancer is a major worldwide health issue with a high prevalence and fatality rate. It is the sixth most prevalent cancer worldwide [1,2]. The prevalence is higher in underdeveloped nations than in developed nations. In India, oral squamous cell carcinoma (OSCC) is the most frequent form of head and neck cancer, accounting for 40-50% of all malignancies [2]. Tobacco and alcohol intake are the most important known risk factors for oral cancer. Recently, infection with high-risk human papillomavirus (HPV) has become evident and has been etiologically linked to the development of oral cancers. HPV is a key risk factor to be ruled out when assessing cases of oral cancer and is highly associated with oral sexual behavior. These viruses are considered carcinogenic infectious agents not only in cervical cancer but also in a small proportion of oral cancers [3]. The most commonly detected high-risk types are HPV16 and HPV18 [2].

In oral cancers, HPV appears as an early initiator of proliferation in the early stages of carcinoma. The viral oncoproteins E6 and E7 associated with HPV cause cancer by binding to and inactivating the tumor suppressor proteins p53 and pRb (retinoblastoma protein). This affects transcriptional cell control, thereby promoting malignant transformation of HPV-infected cells, leading to p16 overexpression, which is easily detected by immunohistochemistry (IHC) [1].

HPV-positive head and neck squamous cell carcinoma (HNSCC) shows a stronger association with sexual behavior compared with HPV-negative HNSCC [4]. Up to 80% of cases of HNSCC are ascribed to tobacco use as the primary cause. Both tobacco and alcohol use act synergistically to enhance the risk of HNSCC. While HPV infection has been linked to a significant increase in HNSCC incidence, declining tobacco use has been associated with a decrease in the prevalence of this cancer [5]. p16 IHC has been used as a biomarker and has been shown to be a good indicator for HPV detection. Several studies have demonstrated the utility of p16 IHC in detecting HPV in oral carcinoma [3]. However, there are limited data in the literature regarding HPV infection in oral carcinoma and its association with histological grading using p16 IHC.

Hence, the present study aims to detect HPV infection in oral carcinoma using p16 IHC and to assess its association with histological grading.

## Materials and methods

This study was a cross-sectional study conducted at the Histopathology Laboratory of KLE’s Dr. Prabhakar Kore Charitable Hospital and Medical Research Center, Belagavi, between January 2021 and December 2021. The sample size was calculated using Cochran’s formula, and a total of 50 cases of oral carcinoma were studied for HPV infection.

Patients older than 18 years with tumors within the oral cavity diagnosed histopathologically as oral carcinoma were included in the study. Cases diagnosed with premalignant lesions or dysplasia were excluded.

All biopsies and resection specimens of oral carcinoma received at the histopathology laboratory were collected, numbered, fixed in 10% formalin overnight, and processed. Paraffin-embedded blocks were prepared, and sections measuring 3-4 µm were cut using a microtome and mounted onto slides. Fifty slides were stained with H&E for histological grading and histopathological evaluation. Another 50 pre-coated poly-L-lysine slides were baked at 37°C overnight and, prior to testing, baked at 60°C for one hour.

After deparaffinization, antigen retrieval was performed using a buffer solution (TRIS buffer + EDTA), and the slides were heated in a pressure cooker for three whistles. IHC was performed using a specific mouse monoclonal antibody to p16 (clone: p16-JC8) and incubated for 45-60 minutes in a closed chamber at room temperature. Carcinoma of the cervix was used as the positive control, while carcinoma of the cervix showing only cytoplasmic staining, as well as slides processed without the use of a primary antibody, served as negative controls.

Both H&E- and p16 IHC-stained slides were evaluated. H&E-stained slides were reported using the modified Broder’s grading system and graded by a pathologist as well differentiated (Grade 1), moderately differentiated (Grade 2), and poorly differentiated (Grade 3), based on the degree of differentiation.

The p16 IHC slides were assessed under an Olympus BX41 microscope (Olympus Corporation, Tokyo, Japan). Selected images were captured using a JENOPTIK SUBRA digital camera with GRYPHAX software (Jenoptik AG, Jena, Germany), and p16 overexpression was interpreted using the criteria shown in Table 1, Table 2, and Table 3.

**Table 1:** Criteria used to interpret the p16 staining pattern/expression Source: [6]

Positive pattern of staining	Negative pattern of staining
Both nuclear and cytoplasmic	Only cytoplasmic staining or complete absence of both nuclear and cytoplasmic staining

**Table 2:** Criteria used to interpret the percentage of tumor cell staining for p16 Source: [6]

Percentage of tumor cell staining	Grade
1-25%	1+
26-50%	2+
50-75%	3+
>75%	4+

**Table 3:** Criteria used to interpret the intensity of p16 staining Source: [6]

Intensity of tumor cell staining	Score
No tumor cells stained	0
Mild/weak/bare	1
Moderate/patchy	2
Strong/diffuse	3

Statistical analysis

The data obtained were entered into Microsoft Excel (Microsoft Corporation, Redmond, WA, USA), analyzed, and expressed as percentages and proportions. Cases showing p16 positivity and its association with histopathological grading were evaluated. A p-value of less than 0.05 was considered statistically significant. IBM SPSS Statistics for Windows, Version 21.0 (Released 2012; IBM Corp., Armonk, NY, USA) was used for the analysis, with the chi-square test applied to assess associations, and a p-value of less than 0.05 was considered significant.

## Results

This study included 50 cases (n = 50) of oral carcinoma. The patients’ ages ranged from 31 to 70 years, with a mean age of 53.78 years, as shown in Table 4.

**Table 4:** Age distribution of patients with OSCC OSCC, oral squamous cell carcinoma

Age groups (years)	Frequency (n = 50)	Percentage (%)
<50	19	38
≥50	31	62
Total	50	100
Mean ± SD	53.78 ± 11.76	

Out of 50 cases, 41/50 (82%) were male, and 9/50 (18%) were female, with a sex ratio of 4.5:1, as shown in Table 5.

**Table 5:** Sex of the patients with OSCC OSCC, oral squamous cell carcinoma

Sex	Frequency (n = 50)	Percentage (%)
Male	41	82
Female	9	18
Total	50	100

The most common site of involvement was the buccal mucosa in 21/50 cases (42%), followed by the tongue in 16/50 cases (32%) and the gingivobuccal sulcus in 6/50 cases (12%), as shown in Table 6 (N = 50).

**Table 6:** Site of involvement of OSCC OSCC, oral squamous cell carcinoma

Site of lesion	Number (n = 50)	Percentage (%)
Alveolus	4	8
Gingivobuccal sulcus	6	12
Buccal mucosa	21	42
Tongue	16	32
Lip	3	6
Total	50	100

**Figure 1:**
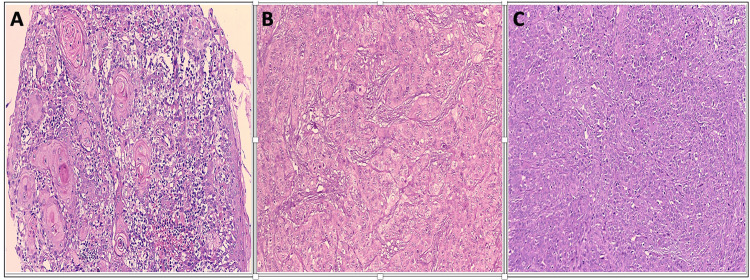
Histopathological evaluation according to the modified Broder’s grading system (A) Well-differentiated OSCC (H&E, 100×). (B) Moderately differentiated OSCC (H&E, 200×). (C) Poorly differentiated OSCC (H&E, 100×). OSCC, oral squamous cell carcinoma

On histopathological evaluation, the most common histological grade observed was moderately differentiated carcinoma in 35/50 cases (70%), followed by well-differentiated carcinoma in 10/50 cases (20%) and poorly differentiated carcinoma in 5/50 cases (10%), as shown in Figure 1 and Table 7.

**Table 7:** Histopathological evaluation according to the modified Broder’s grading system

Histopathological diagnosis	Frequency (n = 50)	Percentage (%)
Poorly differentiated	5	10
Moderately differentiated	35	70
Well differentiated	10	20
Total	50	100

**Figure 2:**
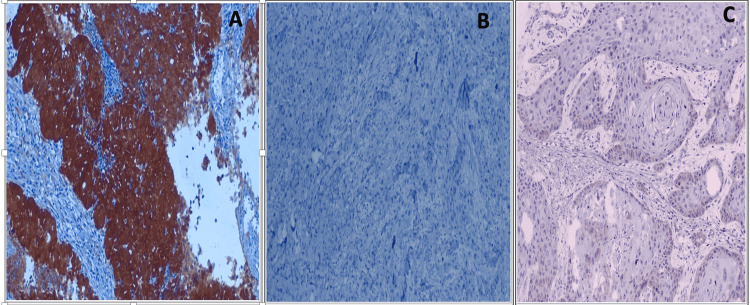
Positive and negative control of p16 staining (A) p16-positive control (IHC, 100X). (B) p16-negative control (IHC, 100X). (C) p16-negative control (IHC, 100X). IHC, immunohistochemistry

The positive and negative controls for p16 overexpression are shown in Figure 2. In the present study, it was observed that out of 50 cases, 24/50 (48%) were positive for p16 (nuclear and cytoplasmic staining), inclusive of all histological grades, while 21/50 cases (42%) showed only cytoplasmic staining and 5/50 cases (10%) showed negative p16 staining, as shown in Table 8.

**Table 8:** Pattern of p16 staining in OSCC cases OSCC, oral squamous cell carcinoma

Pattern of staining	Frequency (n = 50)	Percentage (%)
Nuclear and cytoplasm positive	24	48
Only cytoplasm positive	21	42
Negative staining	5	10
Total	50	100

**Figure 3:**
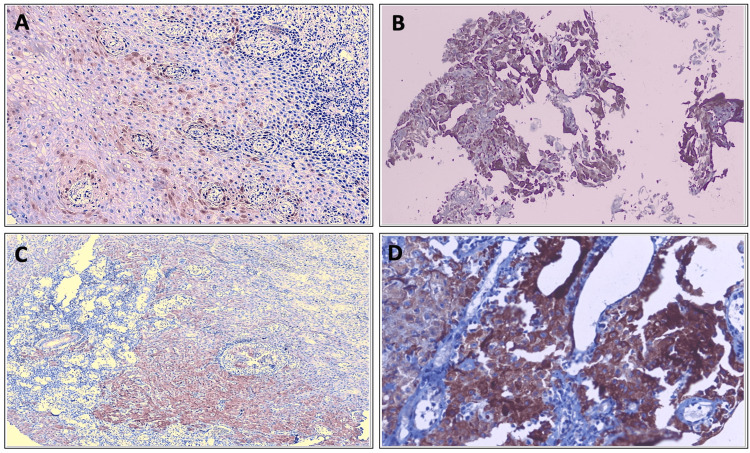
Percentage of positive tumor cells stained in p16-positive OSCC (A) p16 - 1-25% of tumor cell staining (50×). (B) p16 - 25-50% of tumor cell staining (50×). (C) p16 - 50-75% of tumor cell staining (50×). (D) p16 - >75% of tumor cell staining (100×). OSCC, oral squamous cell carcinoma

Among the p16-positive OSCC cases (N = 24), 10/24 cases (41.7%) showed 25-50% of tumor cells stained (Score 2), followed by 8/24 cases (33.3%) with 1-25% staining (Score 1), 5/24 cases (20.8%) with 50-75% staining (Score 3), and only 1/24 case (4%) showed >75% tumor cell staining (Score 4), as shown in Figure 3 and Table 9.

**Table 9:** Percentage of positive tumor cells stained in p16-positive OSCC OSCC, oral squamous cell carcinoma

Percentage (%) of tumor cell staining	Frequency (n = 24)	Percentage (%)
1-25	8	33.3
25-50	10	41.7
50-75	5	20.8
>75	1	4.2
Total	24	100

**Figure 4:**
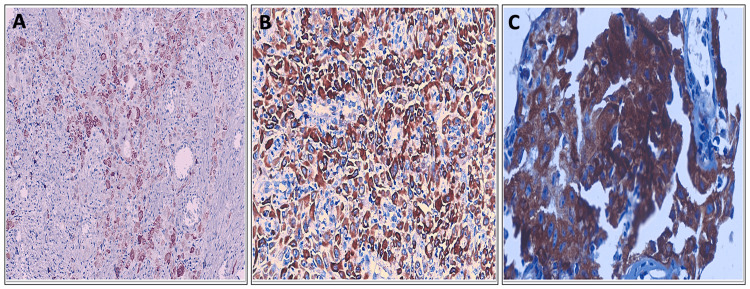
Tumor cell staining intensity grades in p16-positive OSCC (A) p16 - Mild/weak/bare singly dispersed intensity (IHC, 100×). (B) p16 - Moderate/patchy intensity (IHC, 200×). (C) p16 - Strong/diffuse intensity (IHC, 400×). IHC, immunohistochemistry; OSCC, oral squamous cell carcinoma

Among the p16-positive OSCC cases (N = 24), 5/24 cases (20.8%) showed weak, bare, or singly dispersed staining intensity (Grade 1), 16/24 cases (66.7%) showed moderate or patchy staining intensity (Grade 2), and 3/24 cases (12.5%) showed strong or diffuse staining intensity (Grade 3), as shown in Figure 4 and Table 10.

**Table 10:** Tumor cell staining intensity in p16-positive OSCC OSCC, oral squamous cell carcinoma

Intensity	Frequency (n = 24)	Percentage (%)
1	5	20.8
2	16	66.7
3	3	12.5
Total	24	100

IBM SPSS Statistics for Windows, Version 21.0, was used for the analysis, with the chi-square test applied to assess associations, and a p-value of less than 0.05 was considered statistically significant. Among the p16-positive OSCC cases, the association between patient age and the percentage of positive tumor cells was statistically significant (p-value = 0.016; p < 0.05), whereas the association between patient sex and the percentage of positive tumor cells was not statistically significant (p-value = 0.912), as shown in Table 11 (N = 24).

**Table 11:** Association of patient age and sex with the percentage of positive tumor cells among p16-positive OSCC cases OSCC, oral squamous cell carcinoma

Characteristics		Percentage (%) of positive tumor cells (n = 24)				p-Value
		1-25	25-50	50-75	>75	
Age	<50	0 (0.0%)	4 (44.4%)	4 (44.4%)	1 (11.2%)	0.016
	≥50	8 (53.3%)	6 (40.0%)	1 (6.7%)	0 (0.0%)	
Sex	Male	6 (33.3%)	7 (38.9%)	4 (22.2%)	1 (5.6%)	0.912
	Female	2 (33.3%)	3 (50.0%)	1 (16.7%)	0 (0.0%)	

However, the association between HPV infection and histological grading of oral carcinoma among p16-positive cases was not statistically significant (p-value = 0.304), as shown in Table 12 (N = 24). This result may be due to the influence of sample size, study duration, geographic distribution of tumors, differences in antibodies used for staining, and scoring criteria applied.

**Table 12:** Association of HPV infection with the histological grading of oral carcinoma using p16 IHC HPV, human papillomavirus; IHC, immunohistochemistry

Nuclear and cytoplasmic staining of p16	Histological grading (n = 24)			p-Value
	Poorly differentiated	Moderately differentiated	Well differentiated	
Absent	4 (15.4%)	16 (61.5%)	6 (23.1%)	0.304
Present	1 (4.2%)	19 (79.2%)	4 (16.7%)	
Total	5 (10.0%)	35 (70.0%)	10 (20.0%)	

## Discussion

Oral cancer is a major worldwide health issue with a high prevalence and fatality rate [1,2]. According to WHO, current estimates show approximately 657,000 new cases of oral and pharyngeal cancer and over 330,000 deaths each year, with South Central Asia carrying the highest burden due to exposure to risk factors [7,8]. According to Global Cancer Observatory data, the estimated annual incidence of OSCC in 2020 was 377,713 cases worldwide, with Asia reporting the highest number (248,360), followed by Europe (65,279) and North America (27,469). By 2040, the incidence and mortality of OSCC are predicted to increase by up to 40% [9]. In India, OSCC accounts for 40-50% of all malignancies [2].

The primary known risk factors for oral cancer are tobacco and alcohol consumption, which act synergistically to increase risk by up to 35% [10]. Recently, infection with high-risk HPV has been recognized as a key etiological factor in the development of oral cancers. HPV is an important risk factor to consider when assessing cases of oral cancer and is highly associated with oral sexual behavior. These viruses are considered carcinogenic infectious agents not only in cervical cancer but also in a proportion of oral cancers. The most commonly detected high-risk types are HPV16 and HPV18 [2]. In oral cancers, HPV appears in the early stages of carcinogenesis, acting as an initiator of proliferation. HPV-associated cancers affect the cell cycle via the viral oncoproteins E6 and E7, which bind to and inactivate the tumor suppressor proteins p53 and pRb (retinoblastoma protein). This disruption of transcriptional control promotes malignant transformation of HPV-infected cells, leading to p16 overexpression, which is readily detected by IHC [1].

In the present study, patient ages ranged from 31 to 70 years, with a mean age of 54.12 years. Sixty-two percent of cases were over 50 years old, and 38% were below 50 years old. Compared to previous studies by Deng et al., Liang et al., and Pandey et al., the mean ages were 54, 61.6, and 56, respectively [11-13]. The mean age in our study is similar to that reported by Ralli et al. [14]. In our study, male patients accounted for 75% of p16 overexpression, compared to 25% in females, but this difference was not statistically significant. Studies by Liang et al. and Kanyilmaz et al. also found higher p16 overexpression in males, with significant p-values [12,15]. This may be due to the higher prevalence of tobacco use, either chewing or smoking, and alcohol consumption among males. All these etiologies contribute to the development of OSCC.

In our study, the most common site of involvement was the buccal mucosa (50%), and the least common site was the lip (4%). This contrasts with studies by Bai et al., Prakash et al., and Pires et al., which reported the tongue as the most commonly involved site [16-18]. A study by Sudhakaran et al. also identified the buccal mucosa as the predominant site of OSCC, consistent with our findings [19]. This may reflect common forms of smokeless tobacco used in the region, including khaini, gutkha, betel quid with tobacco, and zarda.

WHO reports that nearly 267 million adults (>15 years) in India use tobacco, according to the Global Adult Tobacco Survey India, 2016-17, with smokeless tobacco being the most prevalent form, alongside smoking forms such as cigarettes, bidis, and hookah [20]. Studies by de C. Ferreira C et al. and Tokuzen et al. found very low p16 positivity (10% and 32%, respectively) [21,22], whereas Patil et al. and Azizi et al. reported very high p16 positivity (93% and 87%, respectively) [2,23]. Other studies by Pandey et al., Sudhakaran et al., and Hashmi et al. reported p16 positivity of 60%, 44%, and 50%, respectively, which is concordant with the 48% positivity observed in the present study [13,19,24]. This variability may be influenced by ethnicity, geography, exposure to tobacco and alcohol, frequency of oral sex, and sample size.

Additional factors contributing to variability include differences in the interpretation of IHC results, cutoff values used for assessing intensity, percentage of tumor cells stained, staining patterns, and the types of antibodies used. Studies by Deng et al., Ralli et al., and Meng et al. reported a significant association between HPV and the histological grading of OSCC using p16 (p < 0.05) [11,14,25]. However, in our study, no statistically significant association was found between HPV and histopathological grading using p16 IHC (p = 0.304), consistent with the findings of Pandey et al. and Yuen et al. [13,26]. These discrepancies may be due to differences in sample size, study duration, geographic distribution of tumors, antibodies used, and scoring criteria.

Our study has some limitations. The sample size was small (n = 50), which may have limited the ability to detect a significant association between p16 and histopathological grading of OSCC. In addition, p16-positive cases were not compared with PCR, the gold standard for HPV DNA detection, due to the higher cost.

The strength of this study is that p16 IHC is a useful biomarker, particularly for high-risk HPV infections, making it valuable for evaluating HPV-associated squamous carcinoma. p16 IHC is feasible, cost-effective, reproducible, and more practical than PCR, especially in developing countries, and can inform the development of prophylactic vaccines.

The prognostic significance of p16 expression in OSCC remains unclear. Maléřová et al. reported that p16 positivity may be associated with worse survival and negative prognostic factors, whereas Doll et al. reported better survival with p16 positivity in OSCC [27,28].

## Conclusions

The present study revealed p16 positivity in 48% of OSCC cases, suggesting a low prevalence of HPV-infected OSCC in this region. The study also found no statistically significant association between p16 expression and histopathological grading of OSCC. A comparative study using both p16 IHC and PCR is recommended for future research to achieve a more accurate and definitive diagnosis.
